# Resumed Publication of *Pharmaceuticals* in 2009

**DOI:** 10.3390/ph2010001

**Published:** 2009-04-01

**Authors:** Shu-Kun Lin, Derek J. McPhee

**Affiliations:** Molecular Diversity Preservation International (MDPI), Kandererstrasse 25, Basel CH-4057, Switzerland; Tel. ++41 79 322 3379, Fax: ++41 61 302 8918, Email: lin@mdpi.org; mcphee@mdpi.org

After a five year hiatus, we are pleased to announce the resumption of publication of the MDPI journal *Pharmaceuticals* (ISSN 1424-8247). First launched in 2004, few suitable papers were submitted and only one was published [1], before our limited editorial resources at the time led us to temporarily discontinue publication. Several things have changed since then. First, there has been an explosive growth in the number of manuscripts submitted and published in MDPI’s various current journals [2], whose topics clearly fall within the intended scope of *Pharmaceuticals* and we feel that these manuscripts merit a dedicated forum. Second, the expansion of MDPI, now with Editorial Offices and staff in Basel (Switzerland) and Beijing (China), allows us to provide *Pharmaceuticals*’ authors with all the services they could desire and deserve: a simple manuscript submission process, rigorous peer review, quick revision turnaround, Open Access publication on a new and attractive platform and coverage by all the major abstracting services. In addition, a new Editorial Board comprised of noted academic and industry scientists has been set up for *Pharmaceuticals.* Finally, to better focus the subject matter published in *Pharmaceuticals* on molecular medicines, we have also set up a special section in *International Journal of Molecular Sciences* (IJMS, ISSN 1422-0067) for papers on nutraceuticals or chemopreventives. We look forward to receiving and publishing your papers and as always, we welcome your comments and suggestions.
